# Effects of Stability of Base Pairs Containing an Oxazolone on DNA Elongation

**DOI:** 10.1155/2014/178350

**Published:** 2014-12-07

**Authors:** Masayo Suzuki, Kazuya Ohtsuki, Katsuhito Kino, Teruhiko Kobayashi, Masayuki Morikawa, Takanobu Kobayashi, Hiroshi Miyazawa

**Affiliations:** Kagawa School of Pharmaceutical Sciences, Tokushima Bunri University, 1314-1 Shido, Sanuki, Kagawa 769-2193, Japan

## Abstract

The nucleoside 2,2,4-triamino-5(2*H*)-oxazolone (Oz) can result from oxidative damage to guanine residues in DNA. Despite differences among the three polymerases (Pol *β*, KF exo^−^, and Pol *η*) regarding nucleotide incorporation patterns opposite Oz, all three polymerases can incorporate guanine opposite Oz. Based on *ab initio* calculations, we proposed a structure for a stable Oz:G base pair. Here, to assess the stability of each Oz-containing base pair (Oz:G, Oz:A, Oz:C, and Oz:T) upon DNA replication, we determined the efficiency of Pol *β*-, KF exo^−^-, or Pol *η*-catalyzed primer extension beyond each base pair. With each polymerase, extension beyond Oz:G was more efficient than that beyond Oz:A, Oz:C, or Oz:T. Moreover, thermal denaturation studies revealed that the *T*_*m*_ value for the duplex containing Oz:G was significantly higher than those obtained for duplexes containing Oz:A, Oz:C, or Oz:T. Therefore, the results from *ab initio* calculations along with those from DNA replication assays and thermal denaturation experiments supported the conclusion that Oz:G is the most stable of the Oz-containing base pairs.

## 1. Introduction

**Scheme 1:**
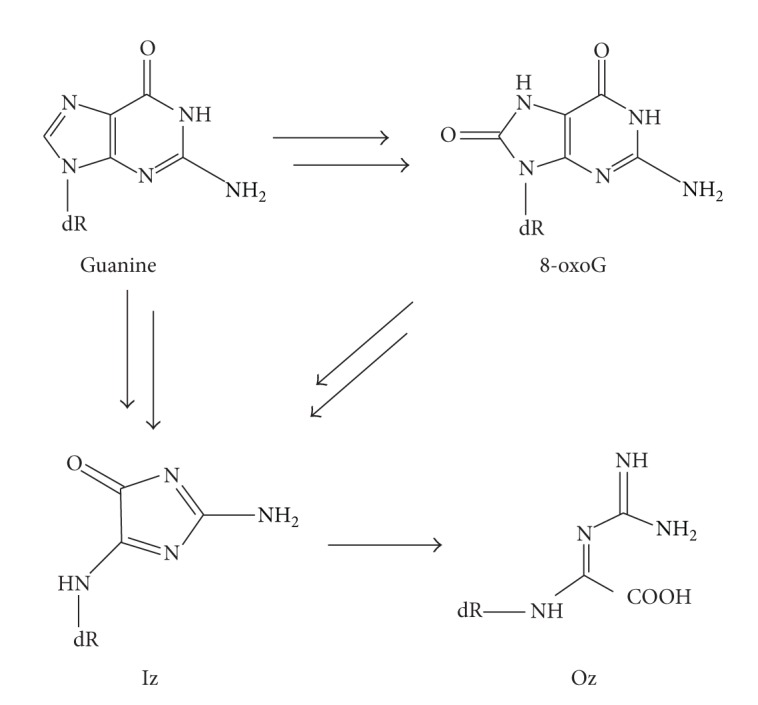
Products of oxidation of guanine and 8-oxoG.

DNA is constantly damaged by various oxidative stresses. Oxidized DNA causes mutations that can lead to aging, carcinogenesis, and other diseases. Guanine has the lowest oxidation potential among the four bases; therefore, it is much more sensitive than A, T, or C to oxidative stresses. G:C to T:A and G:C to C:G transversions are preferentially caused by several oxidative stresses and are observed* in vivo*; for example, G:C-T:A and G:C-C:G transversions caused by passive smoking were detected in codons 12 and 13 of the* K-ras* gene [1]. 8-Oxo-7,8-dihydro-guanine (8-oxoG) is a typical form of oxidative guanine damage (Scheme 1), and 8-oxoG arises under various oxidative conditions. 8-oxoG can pair with adenine but not guanine; therefore, 8-oxoG can generate G:C-T:A transversions [2]. G:C-C:G transversions are assumed to be caused by other forms of oxidative guanine damage.

2,5-Diamino-4*H*-imidazol-4-one (Iz) can be formed from guanine or 8-oxoG under various oxidative conditions (Scheme 1) [3, 4]. Iz and guanine can potentially form base pairing structures that can in turn cause G:C-C:G transversions [5]. However, Iz is slowly hydrolyzed to 2,2,4-triamino-5(2*H*)-oxazolone (Oz); this reaction has a half-life of 147 min under physiological conditions (Scheme 1) [3]. In samples of liver DNA, two to six molecules of Oz are detected per 10^7^ guanine bases [6], and the biological impact of Oz should not be ignored.

**Figure 1:**
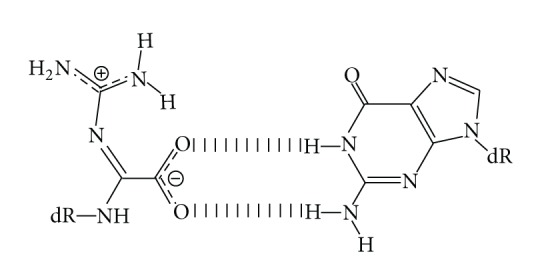
Proposed base pairing between Oz and G.

We previously investigated the incorporation of nucleotides opposite Oz by various DNA polymerases [7, 8]. We found that Pol *α*, *β*, and *ε* each incorporated only guanine opposite Oz; in contrast, Klenow Fragment exo^−^ (KF exo^−^), Pol *γ*, and Pol IV each incorporated either guanine or adenine; Pol *η* incorporated guanine, adenine, or cytosine. That is, incorporation of guanine opposite Oz was common to all DNA polymerases used in this analysis [7, 8], and Oz appears to participate in the generation of G:C to C:G transversions. Based on previous* ab initio* calculations, we predicted that (1) Oz forms a stable base pair with guanine, (2) the Oz:G base pair is planar, and (3) it has two hydrogen bonds (Figure 1) [8–10]. These predictions can explain the observation that guanine is incorporated opposite Oz by each of the polymerases used in the analysis.

Generally, the replicative DNA polymerases have difficulty progressing through distorted DNA, and mismatched purine (Pur):Pur or pyrimidine (Pyr):Pyr base pairs are more distorted and less stable than Pur:Pyr or Pyr:Pur base pairs. Previous simulations of molecular dynamics indicate that DNA containing a mismatch (either G:G, C:C, or C:A) at the primer terminus is distorted, and the hierarchy of the distortions are as follows: G:G > C:C > C:A [11]. Previous experimental findings show that the efficiency of correct nucleotide incorporation is higher with a Pur:Pyr or Pyr:Pur mismatch at a primer terminus than with a Pur:Pur or Pyr:Pyr mismatch [12]. Therefore, the extension activity of DNA polymerase is dependent on the stability of the base pair at the primer terminus.

The aim of this study was to assess the stability of each of the four base pairs (Oz:G, Oz:A, Oz:C, and Oz:T) during DNA replication. For this purpose, we used primer extension assays to measure extension of primers that had an Oz:G, Oz:A, Oz:C, or Oz:T base pair at the primer terminus/extension site; we used each of the three polymerases (Pol *β*, KF exo^−^, and Pol *η*) in these assays to assess consistency of our findings. In addition, thermal denaturation studies can be used to measure the stability of the double-stranded DNA and, by extension, the stability of the base pairs included in duplex. Therefore, we subjected DNA duplex containing an Oz residue to thermal denaturation studies.

## 2. Materials and Methods

### 2.1. Proteins

A human* POLH *(which encodes Pol *η*) cDNA was amplified via PCR from a cDNA template isolated from HEK293 cells; KOD-Plus-Neo (TOYOBO) and two* POLH*-specific primers (XhoI-linked forward primer, 5′-TACTCGAGATGGCTACTGGACAGGATCGAGTG-3′, and XhoI-linked reverse primer, 5′-TACTCGAGCTAATGTGTTAATGGCTTAAAAAA-3′; XhoI sites are underlined) were used for amplification. The human* POLH* cDNA fragment was cloned into the XhoI sites of the vector pET-15b (Novagen) to generate the pET-15b/POLH-NHis_6_ construct, which encoded recombinant human Pol *η* fused to an N-terminal His_6_ tag.

Recombinant, His-tagged human Pol *η* was expressed in the* E. coli *strain Rosetta2 (DE3) pLysS.* E. coli* Rosetta2 (DE3) pLysS cells harboring pET-15b/POLH-NHis_6_ were grown in Luria-Bertani medium supplemented with ampicillin (100 *μ*g/mL) at 37°C with aeration. When cultures reached OD_600_ 0.6–0.8, IPTG (1 mM) was added to the cells to induce expression of recombinant Pol *η*; 3 h after induction, cells were collected by centrifugation. Harvested cell pellets were resuspended in lysis buffer (20 mM sodium phosphate, pH 7.4, 0.5 M NaCl, and 1% TritonX-100). Suspensions were sonicated, and cell lysates were clarified by centrifugation. The resulting supernatants were loaded onto a HisTrap HP 1 mL (GE Healthcare), and each column was washed with Buffer A (20 mM sodium phosphate, pH 7.4, 0.5 M NaCl, and 40 mM imidazole). Bound His_6_-tagged Pol *η* was eluted with Buffer B (20 mM sodium phosphate, pH 7.4, 0.5 M NaCl, and 500 mM imidazole). Fractions containing recombinant Pol *η* were dispensed in small aliquots into storage buffer (20 mM sodium phosphate, pH 7.4, 0.1 M NaCl, 1 mM EDTA, 10% glycerol, 10 mM *β*-mercaptoethanol, and 0.5 mM phenylmethylsulfonyl fluoride) and stored at −80°C.

Recombinant Pol *β* and KF exo^−^ proteins were purchased from CHIMERx and Fermentas, respectively.

### 2.2. Oligodeoxynucleotides

A 30-mer DNA template (5′-CTCATCAACATCTTXAATTCACAATCAATA-3′, where X represents Oz) for polymerase assays and a 9-mer DNA oligomer (5′-TGCTXGCGT-3′, where X represents Oz) for thermal denaturation studies were prepared as described previously [8]. For polymerase assays, the DNA template (5′-CTCATCAACATCTTGAATTCACAATCAATA-3′) and 5′-Alexa680-labeled 16-mer primers (5′-TATTGATTGTGAATTN-3′, where N represents either a C, G, A, or T) were purchased from Japan Bio Services. For thermal denaturation studies, 9-mer DNA oligomer (5′-TGCTNGCGT-3′ where N represents C or T and 5′-ACGCNAGCA-3′ where N represents C, G, A, or T) was synthesized using the standard phosphoramidite method.

### 2.3. Polymerase Assay for Primer Extension

Primer extension assays with all four dNTPs (5 *μ*L) were carried out in mixtures containing the following components: (for Pol *β*) 50 mM Tris-HCl, pH 8.8, 10 mM MgCl_2_, 1 mM DTT, and 400 *μ*g/mL BSA; (for KF exo^−^) 50 mM Tris-HCl, pH 8.0, 5 mM MgCl_2_, 1 mM DTT, and 100 *μ*g/mL BSA; and (for Pol *η*) 40 mM Tris-HCl, pH 8.0, 1 mM MgCl_2_, 10 mM DTT, 250 *μ*g/mL BSA, 60 mM KCl, and 25% glycerol. All reaction mixtures contained 20 nM of the template, 10 nM of each primer, and 100 *μ*M of each of the four dNTPs (dATP, dCTP, dGTP, and dTTP). The concentrations used for each DNA polymerase are specified in the respective figure legends. Reactions were performed at 37°C for 30 min and terminated by adding 5 *μ*L of stop buffer (15 mM EDTA/10% glycerol). Aliquots (2.5 *μ*L) of each reaction were subjected to electrophoresis in a denaturing 16% polyacrylamide gel containing 8 M urea at 30 W for 60 min. The Odyssey Infrared Imaging System (LI-COR) was used to measure signals on gel images.

### 2.4. Polymerase Assay for Incorporation opposite 5′-Neighboring Bases of G or Oz

Reaction mixtures similar to those described for the primer extension assays were used to analyze nucleotide selectivity during nucleotide incorporation opposite the bases adjacent to G or Oz. However, each of these reaction mixtures contained 20 nM of the template, 10 nM of each primer 5′-TATTGATTGTGAATTA(C/G)-3′, and 100 *μ*M of a single dNTP (dCTP, dGTP, dATP, or dTTP).

### 2.5. Thermal Denaturation Studies

Thermal denaturation studies to determine *T*_*m*_ values were conducted with a 9-mer DNA strand (5′-TGCTXGCGT-3′, in which X represents C, T, or Oz) and a complementary 9-mer strand (5′-ACGCNAGCA-3′, where N represents G, A, C, or T). Samples (100 *μ*L) for thermal denaturation studies contained a 1 : 1 molar ratio of a 9-mer DNA strand and the complementary 9-mer strand in 50 mM sodium phosphate, pH 7.0, and 1 M NaCl with a final duplex concentration of 4 *μ*M. Complementary oligomer pairs were allowed to anneal; each sample was heated in water bath to 60°C for 5 min and then slowly cooled to room temperature. Each sample was then covered with 90 *μ*L of silicone oil (Life Technologies) to prevent evaporation by heating; an Ultrospec 3100 pro (GE Healthcare) was used to heat annealed samples from 20°C to 80°C at a rate of 1°C/min. As the samples were heated, the absorbance was monitored at 260 nm. Each of the *T*_*m*_ values was determined from the maximum in the first derivative of the melting curve.

## 3. Results and Discussion

### 3.1. Primer Extension Assay with Pol *β*

We investigate the efficiency of Pol *β*-mediated primer extension past a lesion because Pol *β* incorporates only guanine opposite Oz [8]. Specifically, we designed two 30-mer DNA templates that were identical except that one contained a G and the other contained an Oz; we also designed four different 16-mer primers that differed only in the nucleotide at the 3′ end; this nucleotide would pair with the G or Oz in the template.

**Figure 2:**
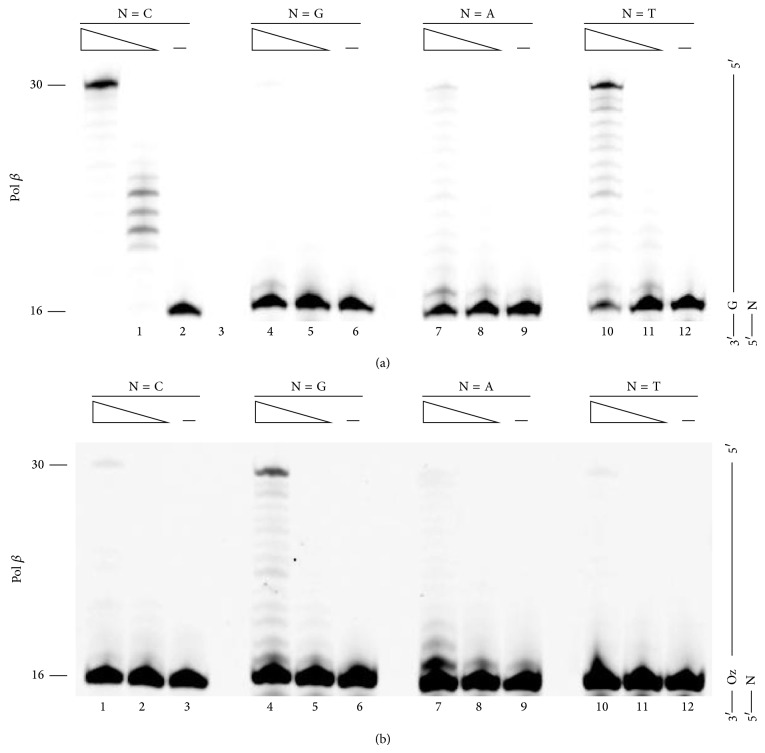
Extension from primer ends by Pol *β*. Each primer contained a different nucleotide at the 3′ end (indicated by N, where N was C, G, A, or T in lanes 1–3, 4–6, 7–9, and 8–12, resp.) opposite G (a) or Oz (b). The amount of Pol *β* was 25 mU in lanes 1, 4, 7, and 10 or 2.5 mU in lanes 2, 5, 8, and 11.

When the template contained an undamaged G, Pol *β* elongated DNA most efficiently from the primer with a C at the 3′-primer terminus; each of the mispaired primer termini resulted in less efficient primer elongation (Figure 2(a), compare lane 1 with lanes 4, 7, and 10). These results confirmed that Watson-Crick base pair is the most stable. A G:T mismatch at the primer terminus allowed for more efficient elongation than did the two other mismatches (Figure 2(a), compare lane 10 with lanes 4 and 7). These results were consistent with the previous finding that Pur:Pyr base pairs are more stable than Pur:Pur base pairs [12].

With the template containing Oz, Pol *β* elongated DNA more efficiently from the primer with a G opposite the Oz (Figure 2(b), compare lane 4 with lanes 1, 7, and 10). Full-length elongation past the Oz:G base pair was at least 8-fold higher than elongation past any other base pair. These results were consistent with the previous finding that Pol *β* incorporates only G opposite Oz [8]. Therefore, we speculated that Pol *β*-mediated incorporation of G opposite Oz could be attributed to the stability of the Oz:G base pair.

Moreover, Pol *β*-catalyzed full-length elongation past Oz:G was more efficient than elongation past either the G:G or G:A mismatch (compare Figure 2(b) lane 4 with Figure 2(a) lanes 4 and 7). These findings indicated that Oz:G was more stable than the Pur:Pur mismatches upon DNA synthesis that was catalyzed by Pol *β*, which has a relatively high fidelity.

### 3.2. Primer Extension Assay with KF exo^−^

**Figure 3:**
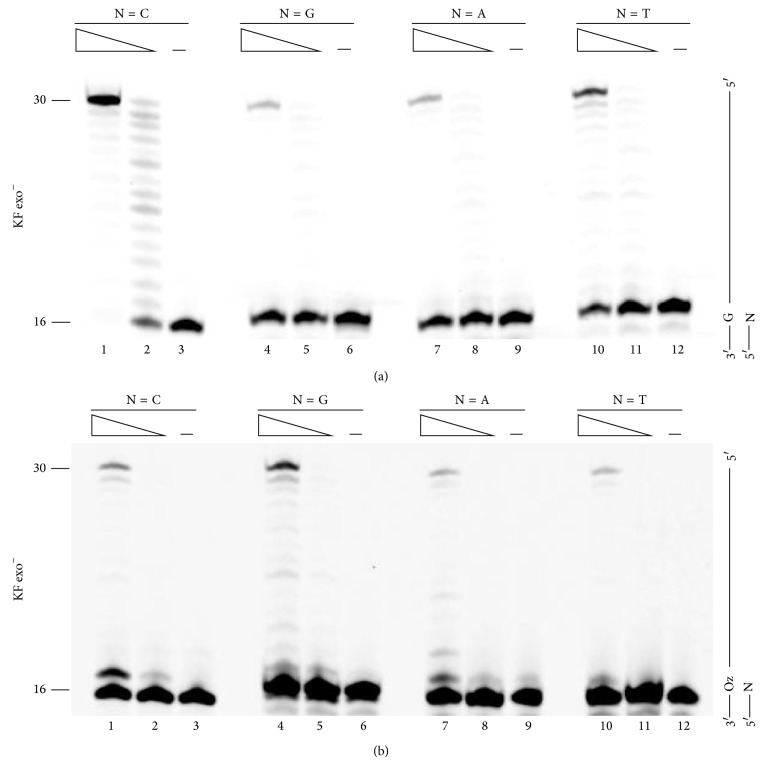
Extension from primer ends by KF exo^−^. Each primer contained a different nucleotide at the 3′ end (indicated by N, where N was C, G, A, or T in lanes 1–3, 4–6, 7–9, and 8–12, resp.) opposite G (a) or Oz (b). The amount of KF exo^−^ was 250 *μ*U in lanes 1, 4, 7, and 10 or 25 *μ*U in lanes 2, 5, 8, and 11.

Next, we examined the ability of KF exo^−^ to extend primers past various primer-template base pairs (Figure 3). KF exo^−^, like Pol *β*, extended primers past G:C more efficiently than past any other base pair (Figure 3(a), compare lane 1 with lanes 4, 7, and 10). Additionally, the order of base pairs with regard to efficiency of extension past a 3′-primer terminus was the same as that for Pol *β*.

Surprisingly, although KF exo^−^ incorporated both G and A opposite Oz [8], a primer containing Oz:G at the 3′-terminus was elongated up to the full length most efficiently (Figure 3(b) lane 4). In addition, the extension efficiency beyond Oz:G was ≥2-fold higher than that beyond Oz:C, Oz:A, or Oz:T (Figure 3(b), compare lane 4 with lanes 1, 7, and 10). This finding indicated that Oz:G was more stable than Oz:A upon primer extension; moreover, this difference in base pair stability was consistent with our previous* ab initio* calculations, which indicated that Oz:G is more stable than Oz:A [8].

### 3.3. Primer Extension Assay with Pol *η*

**Figure 4:**
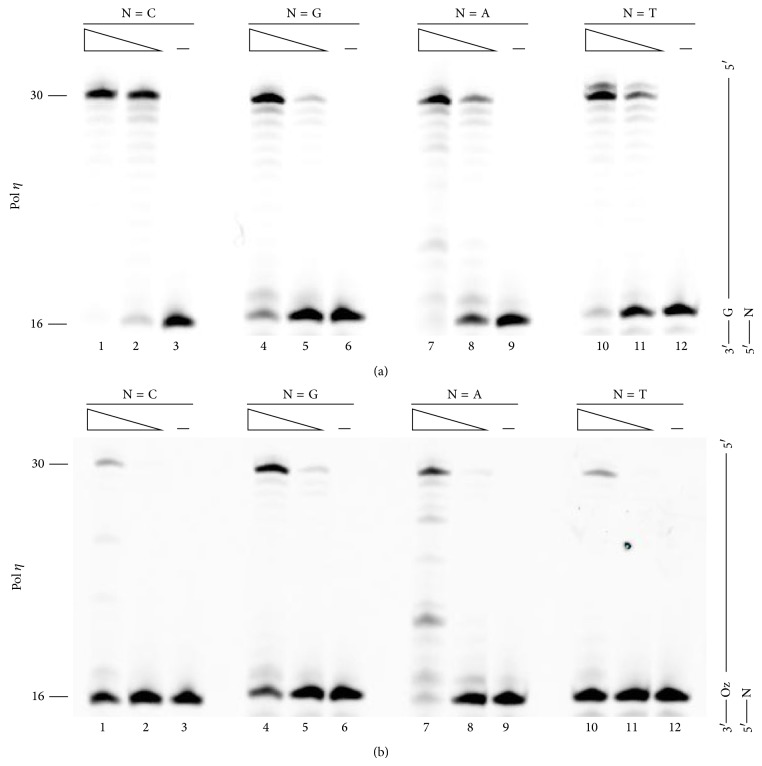
Extension from primer ends by Pol *η*. Each primer contained a different nucleotide at the 3′ end (indicated by N, where N was C, G, A, or T in lanes 1–3, 4–6, 7–9, and 8–12, resp.) opposite G (a) or Oz (b). The amount of Pol *η* was 11.5 ng in lanes 1, 4, 7, and 10 or 1.15 ng in lanes 2, 5, 8, and 11.

As with Pol *β* and KF exo^−^, we analyzed the efficiency of primer extension efficiency past G or Oz with Pol *η* (Figure 4). Pol *η*, like Pol *β* and KF exo^−^, extended primers most efficiently from a C at the 3′-primer terminus (Figure 4(a), compare lane 1 with lanes 4, 7, and 10) whereas, unlike Pol *β* or KF exo^−^, the extension efficiency from a mispaired primer terminus (G:G, G:A, or G:T) was 50% or more of that past a G:C base pair (Figure 4(a), lanes 4, 7, and 10). This finding was unsurprising given that Pol *η* catalyzes error-prone replication of undamaged DNA [13–15].

Our previous study showed that Pol *η* was equally able to incorporate G, A, or C opposite Oz [7], and Pol *η* is known to be error-prone polymerase [13–15]. Nevertheless, the extension beyond Oz:G was about 2-fold more efficient than that beyond Oz:C, Oz:A, or Oz:T (Figure 4(b), compare lane 4 with lanes 1, 7, or 10). This result showed that Oz:G is more stable than each other Oz base pair; this finding was consistent with the findings for Pol *β* or KF exo^−^.

### 3.4. The Thermal Stability of the DNA Duplex Containing the Oz Lesion

Furthermore, the *T*_*m*_ values for duplexes containing Oz:G, Oz:A, Oz:C, or Oz:T were analyzed via thermal denaturation studies. However, the amount of oligonucleotide used in the polymerase reactions was insufficient to perform thermal denaturation studies. The yields from DNA synthesis of DNA oligonucleotides containing Oz usually increase as the length of the oligonucleotide decreases; therefore, we used short sequences that differed from those used in the primer extension reactions.

**Figure 5:**
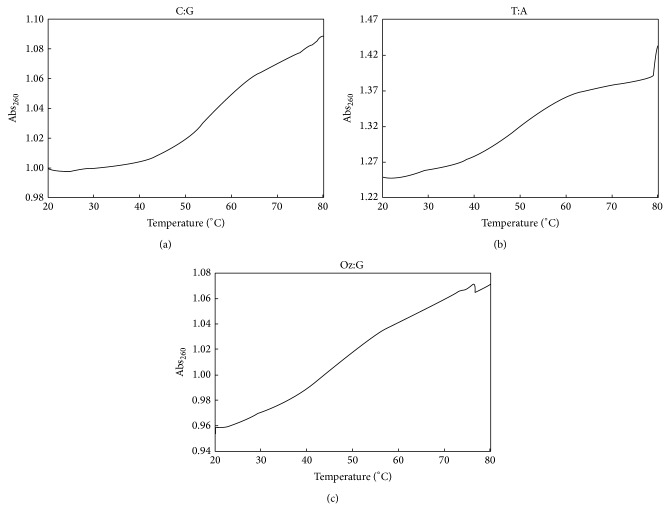
Melting curves of (a) C:G, (b) T:A, and (c) Oz:G 9-mer DNA duplexes at 4 *μ*M duplex concentration.

The *T*_*m*_ value for DNA duplexes containing C:G was 55.1°C (Figure 5(a)) and that for a duplex containing T:A was 48.9°C (Figure 5(b)). This finding showed that C:G was more thermodynamically stable than T:A, which is a well-established fact commonly attributed to the difference in the number of hydrogen bonds forming the respective base pairs.

The *T*_*m*_ value for DNA duplex containing Oz:G was 45.7°C (Figure 5(c)). Meanwhile, *T*_*m*_ values for DNA duplexes containing Oz:A, Oz:C, or Oz:T could not be determined because they were apparently below the detection limit (*T*_*m*_ < 40.0°C). Therefore, the Oz:G base pair was significantly more thermodynamically stable than the Oz:A, Oz:C, or Oz:T base pair.

### 3.5. Nucleotide Selectivity during Incorporation opposite 5′-Neighboring Bases of G or Oz

Findings from the primer extension assays revealed that for Pol *β*, KF exo^−^, or Pol *η* extension beyond Oz:G was more efficient than extension beyond any other base pair involving Oz. However, it is important that in cases of lesion bypass nucleotides are correctly incorporated beyond the lesion. To determine whether Pol *β*, KF exo^−^, Pol *η*, or each of these polymerases can accurately incorporate nucleotides after bypassing a Oz:G lesion, we analyzed nucleotide incorporation opposite the sequence TT immediately adjacent to and 5′ of G or Oz. As with the previous primer extension assays, we used each polymerase individually along with the optimal primer-template combination (G:C or Oz:G at the 3′-primer terminus) for efficient extension.

**Figure 6:**
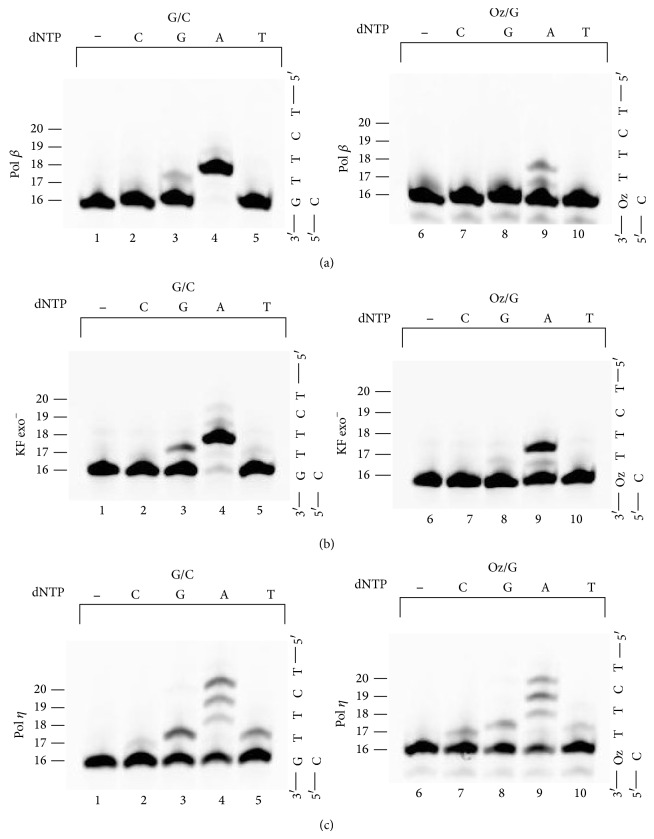
Nucleotide incorporation opposite the bases adjacent to G or Oz by Pol *β* (a), KF exo^−^ (b), or Pol *η* (c). Left panels of (a), (b), and (c) show the control, which was extension of primers containing C opposite an undamaged G in the template. Right panels of (a), (b), and (c) show the extension of primers containing G opposite Oz in the template. The amount of Pol *β* or Pol *η* was 25 mU or 11.5 ng; the amount of KF exo^−^ was 25 *μ*U for the left panel or 250 *μ*U for the right panel.

When the template contained an undamaged G, Pol *β* and KF exo^−^ almost always incorporated two adenines opposite the TT bases that were immediately adjacent to the G in the template strand (Figures 6(a) and 6(b), lane 4); however, each enzyme did incorrectly incorporate some guanine (Figures 6(a) and 6(b), lane 3). Pol *η*, unlike Pol *β* or KF exo^−^, incorporated not only four adenines but also a guanine and a thymine (Figure 6(c), lanes 3–5), owing to the error-prone replication that is a characteristic of Pol *η* [15].

With the Oz template, Pol *β* and KF exo^−^ each incorporated two adenines opposite the sequence TT that were adjacent to Oz in the template (Figures 6(a) and 6(b), lane 9); however, this incorporation was less efficient than that with the template that contained G instead of Oz. Pol *η* incorporated four adenines, a guanine, and a thymine (Figure 6(c), lanes 8–10); however, these incorporation efficiencies with the Oz-containing template were lower than those with the G-containing template. Notably, Pol *η*, unlike Pol *β* and KF exo^−^, incorporated a cytosine opposite the T neighboring Oz (Figure 6(c), lane 7). Thus, Pol *β* and KF exo^−^ can accurately incorporate the correct nucleotides after having passed beyond an Oz in a template; in contrast, Pol *η* was error prone with regard to the nucleotides incorporated beyond Oz to the same degree as with incorporation beyond a G.

## 4. Conclusions

Our previous studies show that Pol *β*, KF exo^−^, and Pol *η* differ with regard to the pattern of nucleotide insertion opposite Oz. Specifically, Pol *β* incorporates only G; KF exo^−^ incorporates either G or A; and Pol *η* incorporates G, A, or C [7, 8]. Based on* ab initio *calculations, we predicted that only G would form a stable base pair with Oz [8–10]. Here, we try to clarify the difference between Oz:G, Oz:A, Oz:C, and Oz:T base pairs with regard to stability during DNA replication with Pol *β*, KF exo^−^, or Pol *η*.

We found that, for each polymerase (Pol *β*, KF exo^−^, or Pol *η*), the efficiency of extension beyond Oz:G was higher than those for extension beyond Oz:C, Oz:A, or Oz:T. Furthermore, Oz:G was significantly more thermodynamically stable than Oz:A, Oz:C, or Oz:T based on determination of *T*_*m*_ values for duplexes containing only one of the base pairs.

Therefore, we demonstrated that Oz:G was a stable base pair in experiments involving DNA replication and thermal denaturation. These conclusions were consistent with our previous* ab initio *calculations, which also indicated that Oz:G is more stable than Oz:A. Specifically, the results of this study reinforce the importance of Oz in oxidative guanine damage involving G:C-C:G transversions.
